# Measuring Nepotism through Shared Last Names: The Case of Italian Academia

**DOI:** 10.1371/journal.pone.0021160

**Published:** 2011-08-03

**Authors:** Stefano Allesina

**Affiliations:** Department of Ecology and Evolution, Computation Institute, University of Chicago, Chicago, Illinois, United States of America; IUMSP, CHUV/University of Lausanne, Switzerland

## Abstract

Nepotistic practices are detrimental for academia. Here I show how disciplines with a high likelihood of nepotism can be detected using standard statistical techniques based on shared last names among professors. As an example, I analyze the set of all 61,340 Italian academics. I find that nepotism is prominent in Italy, with particular disciplinary sectors being detected as especially problematic. Out of 28 disciplines, 9 – accounting for more than half of Italian professors – display a significant paucity of last names. Moreover, in most disciplines a clear north-south trend emerges, with likelihood of nepotism increasing with latitude. Even accounting for the geographic clustering of last names, I find that for many disciplines the probability of name-sharing is boosted when professors work in the same institution or sub-discipline. Using these techniques policy makers can target cuts and funding in order to promote fair practices.

## Introduction

The Bernoulli family, with eight world-famous mathematicians spanning three generations, has left an indelible mark on the way we understand mathematics and physics. Marie Curie won two Nobel prizes for physics and chemistry, her husband Pierre sharing that for physics. Their daughter, Irene Joliot-Curie shared another Nobel prize with her husband. These exceptionally talented families are extremely rare, and in fact in academia the practice of hiring and collaborating with close relatives is generally labeled with the negative term “nepotism” (from *nepos, nepotis* - nephew or grandson in Latin). The idea of nepotism goes back to the Middle Ages, when Popes (who did not “officially” have children) used to nominate their nephews for important posts in the Catholic Church. From the eleventh century until 1692, when the practice was outlawed, it was customary for the Pope to nominate at least one relative to the rank of cardinal (*cardinalis nepos*). Ever since, the word described the practice of favoritism toward close relatives regardless of their merit. Nepotism could be a serious problem in academia, especially in those systems in which career advancements are based on seniority rather than achievements: in these settings, holding a position guarantees career advancements, incentivizing illegal hiring practices. Nepotism clashes with the social norms at the base of science. In particular, it goes against what Merton describes as “Universalism” – research (and researchers) is judged by preestablished impersonal criteria: “Universalism finds further expression in the demand that careers be open to talent” [1].

In Italy, nepotism is perceived as a cancer that has metastasized, invading many segments of society, including academia [2], [3]. The figure of the “barone” (baron), the all-powerful senior professor who can, with a stroke of the pen, make or destroy careers, has permeated popular culture and is frequently represented in novels and movies. Nepotistic practices are especially damaging in a situation in which there are very few new positions (e.g. in Italy, for several years, all academic hires were put on hold). Despite legislative efforts aimed at eradicating nepotism, the general perception is that the practice is alive and well [4]. The more blatant cases have gained the attention of the public, but the magnitude of the problem is unknown, as all the evidence is anecdotal.

Recently, Durante *et al.* [5] performed the first large-scale survey of co-occurrence of last names among Italian academics, and compared it with detailed geographical data on last name frequency. Their analysis showed that the degree of homonymity in academia is much higher than expected at random, especially in some disciplines and institutions. Moreover, they showed that a high degree of homonymity negatively correlates with several indices of academic performance. Although sharing last names does not necessarily imply family affiliation, it can be used as a proxy for nepotistic relations. If anything, the number of cases is going to be largely underestimated, as in Italy women maintain their maiden names, and children take their father's last name. Thus, using last names one can detect nepotism associated with father-child and inter-sibling relations, but not mother-child cases and those involving spouses. Considering that in the sporadic documented cases the majority of hires involves spouses, and that women constitute about a third of the professors, one can conclude that such an analysis can detect roughly half of the cases of nepotism within the immediate family, not to mention lovers, domestic partners, pupils and more distant relatives.

The objective of this work is to show how standard statistical analysis can identify areas with high likelihood of nepotism, and how, using statistical models, policy-makers can focus their attention on particular disciplinary sectors and geographic locations. The identification of areas of intervention is but a first step toward addressing nepotistic practices.

## Results

The dataset analyzed here is a list of all the 61,342 professors in Italy, including their first and last name, institution (94 in total), department and discipline. The dataset includes only tenured academics (equivalent ranks of assistant, associate and full professors), and it does not include temporary workers. In Italy, each academic has to declare a macro and micro disciplinary sector. There are 28 macro-sectors, further divided into 370 micro-sectors. For example, *BIO07* stands for Biological Sciences (*BIO*) - Ecology (*07*). The dataset I gathered from the Ministry of Education (downloaded on October 8, 2010 from the website http://cercauniversita.cineca.it managed by a consortium of 43 universities and the Ministry of Education, University and Research), after filtering out two incomplete records encompassed 61,340 professors spanning all sectors.

The dimension of macro-sectors is highly heterogeneous, the largest being *MED* (Medical Sciences) with 10,783 researchers and the smallest being *M-EDF* (Physical Education) with 138 academics. Also the micro-sectors vary dramatically in size, with one sector containing only one researcher (*L-FIL-LET03*, Italian, Illiric and Celtic Phylology) and one having 1,020 professors (*MED09*, Internal Medicine).

The 61,340 records analyzed contain 27,220 unique last names, of which only four are shared by 100 people or more (Rossi, with 255 academics; Russo, 153; Ferrari, 110; Romano, 100). 17,274 names are associated with only one academic, 4,583 names are shared by two researchers and 1,903 by three. Rare names therefore constitute the bulk of all the names. It is quite easy to determine the gender of the academics using their first name, given that very few Italian names are shared by both genders. In the set, I found 21,057 women, representing 34.33% of the total.

First, I wanted to repeat the previous analysis [5] using a much simpler index. Instead of accounting for couples, triplets and so forth of repeated names in each discipline/geographic area [5], I wanted to build a simple index that is a proxy for the likelihood of nepotistic practices within a discipline (Materials and Methods). What I wanted to ask is whether each discipline displays few distinct last names compared to what might be expected at random. Take for example the medical sciences. There are 10,783 researchers working in this field, accounting for 7,471 distinct names. Is this number small or large? In order to address this question, one would have to create a probability distribution function for the number of names in a sample of equal size taken from the entire Italian population. This is quite difficult, as it would require precise information on the frequency of Italian last names, and their geographic distribution. Because the number of Italian academics is quite large (representing 0.1% of the whole population), one can simplify the problem and ask how probable it is to find more than 7,471 names in a sample of 10,783 taken from the whole set of Italian professors without repetition. In this way, the problem of dealing with last names with different frequencies is accounted for. Although a formula to exactly compute the probability based on a multivariate hypergeometric distribution is known [6], [7], the large sample and population sizes render it inapplicable due to computational constraints. Therefore, I used Monte Carlo methods and computed an approximate 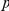-value measuring how probable it is to find a smaller number of distinct names in 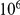 random drawings from the whole set of professors. In the case of medical sciences described above, I never observed an equal or lower number of distinct names out of the million drawings: the paucity of names is extremely unlikely to be observed at random, indicating a very high likelihood of nepotistic practices.

In Table 1 I report, for each macro-sector, the number of academics, number of last names, the expected number of last names from Monte Carlo simulations and the approximated probability of observing a lower or equal number of last names in the sample. In 9 sectors I found 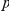-values smaller than 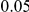, representing fields with high probability of nepotism. These fields include exactly 32,000 researchers: the majority of Italian academics (52.17%) work in disciplines that display a number of names much smaller than expected.

**Table 1:** Likelihood of nepotism for macro-sector.

Macro-sector	People	Names	Expected	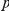 -value	Distance	Institution	Micro	Latitude
Industrial Engineering	3180	2691	2759.4	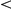 10 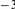	− (***)	+ (***)	+ (*)	− (***)
Law	5144	4031	4207.7	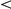 10 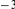	− (***)	+ (***)	+ (**)	− (***)
Medical sciences	10783	7471	7783.2	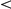 10 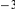	− (***)	+ (***)	+ (***)	− (***)
Geography	377	359	368.3	0.004	− (**)	+ (NS)	+ (NS)	− (***)
Pedagogy	675	634	648.8	0.005	− (***)	+ (***)	+ (*)	− (***)
Agriculture	2345	2058	2095.8	0.007	− (***)	+ (***)	+ (**)	− (***)
Civil Engineering	3836	3206	3259.1	0.008	− (***)	+ (***)	+ (*)	− (***)
Mathematics	2531	2214	2246.5	0.024	− (***)	+ (***)	+ (***)	− (***)
Chemistry	3129	2686	2719.8	0.039	− (***)	+ (***)	+ (NS)	− (***)
History	1453	1329	1346.1	0.054	− (**)	+ (NS)	− (NS)	− (NS)
Earth sciences	1196	1107	1120.8	0.065	− (***)	− (NS)	+ (NS)	− (**)
Philosophy	1125	1045	1057.7	0.071	− (**)	+ (**)	+ (NS)	− (***)
Statistics	1212	1123	1134.9	0.097	− (***)	+ (**)	− (NS)	− (***)
Political sciences	1792	1622	1636.6	0.124	− (***)	+ (*)	− (NS)	− (NS)
Veterinary	847	800	807.0	0.152	− (***)	+ (***)	+ (***)	− (***)
Life sciences	5140	4180	4204.9	0.179	− (***)	+ (***)	+ (***)	− (***)
Informatics	834	789	795.2	0.182	− (***)	+ (NS)	+ (NA)	+ (NS)
Physics	2472	2187	2198.9	0.232	− (***)	+ (***)	− (NS)	− (***)
Economics	3806	3221	3236.6	0.242	− (***)	+ (***)	+ (***)	− (***)
Philology	1780	1618	1626.4	0.254	− (***)	+ (**)	+ (NS)	− (***)
Physical education	138	136	136.8	0.346	− (NS)	− (NS)	− (NS)	− (NS)
Electronic Engineering	2089	1881	1885.4	0.386	− (***)	+ (**)	+ (NS)	− (*)
Art history	815	776	777.8	0.407	− (NS)	+ (NS)	− (NS)	− (NS)
Archeology	704	678	675.7	0.692	− (***)	+ (NS)	+ (NS)	− (NS)
Near eastern studies	317	312	310.8	0.738	− (NS)	− (NS)	− (NS)	− (NS)
Psychology	1252	1176	1170.3	0.754	− (***)	+ (NS)	− (NS)	+ (NS)
Linguistics	2173	2000	1954.8	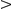 0.999	− (**)	+ (*)	+ (NS)	− (***)
Demography & ethnology	195	195	192.6	1.000	+ (NS)	+ (NS)	+ (NA)	+ (NS)

For each macro-sector, I report the results from Monte Carlo simulations: number of researchers in the discipline (“People”), the number of distinct last names (“Names”), the expected number of last names (“Expected”), and the associated 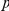-value, measuring how probable it is to find an equal or lower number of names at random. I also report the results from the logistic regression models: effects and the statistical significance of geographic distance (“Distance”), sharing the same institution (“Institution”), sharing the same micro-sector (“Micro”) and the average latitude (“Latitude”) on the probability that two researcher in any discipline have the same name. Magnitude of the coefficients and probabilities are reported in the Supplementary Tables 3, 4, 5, 6 in Supporting Information S1. Significance levels“***” (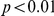), “**” (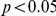), “*” (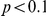), NS (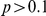), NA (no data available for the coefficient).

The procedure was repeated for the 370 micro-sectors, with similar results (Supplementary Table 2 in Supporting Information S1), and with the same macro-sectors displaying significant departures from expectation. In detail, 45 micro-sectors had 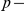
values lower than 0.05 (Supplementary Table 1 in Supporting Information S1). The five disciplines displaying the highest fraction of significant micro-sectors were Pedagogy (3 micro-sectors out 4 with 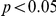), Geography (1 out of 2), Medicine (15 out of 50), Mathematics (2 out of 9) and Civil Engineering (4 out of 22) (Supplementary Tables 1 and 2 in Supporting Information S1). All these disciplines were present in the nine yielding significant results above.

Having established the high degree of nepotism in Italian academia, I took a modeling approach: what is the probability that two researchers share the same last name given some of their characteristics? One can envision a network in which two nodes (academics) are connected if they share a last name. I used logistic regression models to assess the effect of geography, institution, micro-sector and latitude on the probability of connection between nodes. The rationale is that two academics are more likely to share last names if they are geographically closer. This naturally arises from the geographical distribution of last names [8].

Given this baseline “geographic model”, I tested whether the effect of being in the same micro-sector enhanced the probability of sharing the last name. Also, I tested if working in the same institution further increased the probability of last name-connection beyond the geographic effect (belonging to the same institution implies geographic proximity). Finally, I tested if there was a latitudinal effect: does the probability of sharing last names increase when moving from north to south? Previous research found significant differences between the north and south of the country for a variety of statistics, including for example infant mortality, life expectancy, incidence of organized crime [9] and even suicide rate [10]. I therefore tested the effects of the average latitude of the two professors on the connection probability.

**Figure 1:**
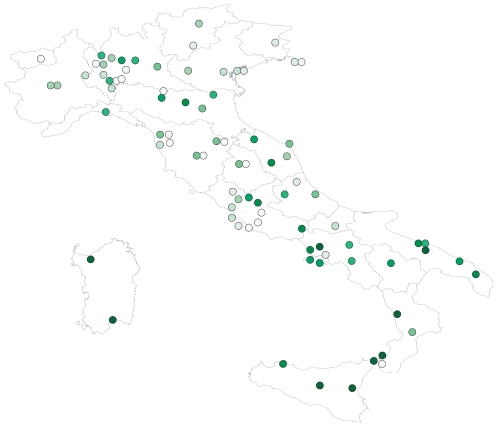
Frequency of last name-sharing in Italian Universities. For each institution (84, as I excluded on-line Universities), I computed the frequency by dividing the number of pairs of professors sharing the last name by the total number of possible pairs. I arrange in a circle the institutions based in the same city (e.g., 9 Universities in Roma). Darker shades stand for higher frequencies. The frequency of last name-sharing for each institution is reported in Supplementary Table 7 in Supporting Information S1.

I report the sign and significance level of all these factors in Table 1. In 24 disciplines out of 28, distance had a highly significant effect: being closer geographically increased the probability of connection. This probability was further increased by affiliation to the same institution in 16 cases. Belonging to the same micro-sector enhanced the probability of connection in 7 cases. Finally, there was a strong north-south gradient in 18 disciplines. Notably, all the disciplines being detected as problematic using 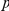-values were also associated with highly significant effects of all the covariates examined, with the exception of Geography (no significant effects of institution and micro-sector) and Chemistry (no effect of micro-sector). In Figure 1, I show the relative frequency of name-sharing within institutions, with higher frequencies concentrated in the south.

## Discussion

Analyzing nepotism directly would require access to sensitive data and a detailed investigation of hiring practices in academia, which in Italy are strongly regulated by law. Here I took a different approach and tested whether the diversity of last names displayed by the various disciplines is lower than expected at random. Clearly, the results can only suggest, but not prove, which disciplines are likely to be impacted by nepotism.

Sharing last names does not necessarily imply family affiliation, and this is why I adopted the Monte Carlo sampling routine. In this way, I accounted for the fact that some names are common and others (the majority) are rare. Note however that analyzing last names excludes cases of nepotism of the mother-child type (as women maintain their maiden name, while children take the father's last name) as well as cases involving spouses or partners. Thus, my analysis should greatly underestimate the real level of nepotism in Italian academia.

Italy has an elongated geography and two main islands, as well as mountain ranges dividing the regions. For this and historical reasons, last names present considerable spatial clustering. Analyzing disciplines, which have a quite uniform spatial distribution, attenuates this problem. In the logistic models, where I tested spatial effects, I first introduced a “geographic” term accounting for the fact that the probability of name-sharing is expected to decrease with geographic distance.

There could be “positive” explanations for the trends emerged in the analysis. For example, the so called “human-capital transfer” [11] could be at play: parents would provide a suitable intellectual environment for children, transferring knowledge, passions, social ties and a view of the world that would result in a higher chance of choosing the parents' careers (occupational following). Although this must certainly play a role, it can hardly explain the extremely low probabilities that emerged from the analysis. Also, given that in Italy all academics share basically the same salaries and duties regardless the discipline and institution, the same “academic lifestyle” is guaranteed regardless the disciplinary choice. Thus, one would expect children to “drift” toward related disciplines (requiring the same knowledge and skills) as well as remaining in the same exact field of their parents, reducing the “nepotistic signature” in the data.

Other limitations of the study are statistical. Given that I performed several tests, there is the risk of introducing false positives due to multiple comparisons. Typically, one would take recourse to Bonferroni's or similar corrections to account for multiple hypotheses testing. However, these methods entail considerable loss of power, as they are rooted in the number of tested hypotheses: if one is testing 370 micro-sectors, a significance level of less than 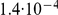 should be used to guarantee an overall significance level of 0.05 for the tests (using Bonferroni's correction). Using these restrictive techniques, only the macro and micro-sectors for which I did never observe a lower number of names out of a million drawings could be considered significant (3 macro, 7 micro).

Similar problems are found in the literature on genetic screenings, where the effects of hundreds or thousands of genes are routinely tested. A useful concept taken from this literature is that of a 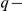
value [12]. This value specifies the expected proportion of “false discoveries” when all the tests resulting in a 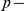
value lower than 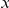 are called significant. I set the 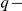
value to 0.05 (i.e. I wanted to keep the expected proportion of false positives under 5%), and I found that all the disciplines with 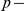
value 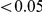 fell in this region. Thus, less than 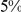 of the nine significant macro-disciplines are likely to be a false positive, confirming the results obtained above. A different outcome is obtained for the micro-sectors, because of their small size and the large number of sub-disciplines: in order to keep a 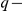
value of 0.05, I would have to call significant only the top 15 micro-sectors (instead of 45). Calling all the tests with 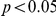 significant, would yield a 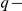
value of 0.37: of these 45 sub-disciplines, 16.65 are likely to be false positives.

Applying the same method to the 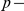
values obtained for the logistic regressions confirms that the significant results for the coefficients of geographic model, those regarding institutions and latitude are unlikely to be false positives (in all cases 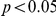 is associated with 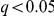). Analyzing shared micro-sectors, however, shows that only 7 of the 10 test for which 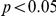 have 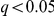. Calling all the 10 values with 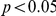 significant, yields an expected proportion of 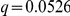.

Both types of analysis (Monte Carlo and logistic regression) showed the same results: the paucity of names and the abundance of name-sharing connections in Italian academia are highly unlikely to be observed at random. Many disciplines, accounting for the majority of Italian academics, are very likely to be affected by nepotism. There is a strong latitudinal effect, with nepotistic practices increasing in the south. Although detecting some nepotism in Italian academia is hardly surprising [2], [3], the level of diffusion evidenced by this analysis is well beyond what is expected.

Concentrating resources in the “healthy” part of the system is especially important at a time when funding is very limited and new positions are scarce: two conditions that are currently met by the Italian academic system. Moreover, promoting merit against nepotistic practices could help stem the severe brain-drain observed in Italy [13], [14].

A few of the main pitfalls of the Italian legislation on academic hiring that have led to this situation are: a) all positions are (in practice) tenured: thus, being hired ensures a position for life; b) disciplinary sectors are independent, self-referential entities: the large number of micro-sectors makes them prone to colonization by few professors pushing their agenda; c) the examiners evaluating the candidates for academic positions have no personal incentive to promote merit, as they do not have to pay the consequences of erroneous choices and do not benefit from hiring good researchers.

In December 2010, the Italian Parliament approved a new law for the University. Among other things, the new law forbids the hiring of relatives within the same department and introduces a probation period before tenure. The analysis conducted here should be repeated in the future, as the results could provide an assessment of the efficacy of the new law.

This analysis can be applied to different countries and types of organizations. Policy-makers can use similar methods to target resources and cuts in order to promote fair practices.

## Materials and Methods

### Monte Carlo Drawings

Durante *et al.* [5] analyzed the frequency of last names of Italian academics using a “homonimity index” accounting for deviance in the frequency of last names from that of the population located in the same geographic area. Therefore, building the index requires sophisticated data on the geographic frequency of last names in the Italian population. Moreover, the index implicitly accounts differentially for cases of “concentrated” nepotism (one family with many professors in the same academic unit) and “diffused” nepotism (many families with a few professors each). Because a) the data on the geographic distribution of names could not be available for many real-world cases and b) generally, we do not have an *a priori* preference between “diffused” and “concentrated” nepotism (both types seem equally damaging for academia), I addressed the problem in a different way.

I constructed an index for the level of nepotism in each discipline. Each discipline contains 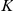 researchers displaying 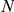 distinct last names. From the whole population of 61,340 records, I extracted 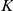 records at random 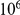 times. Records were sampled without repetition. I then counted the number of distinct names 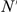 present in the samples. I recorded the expected number of names (averaging the number of names in the samples) and the probability of observing a number of names 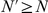. This 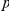-value is what I report in the Tables. Low values stand for high probability of nepotism. Because drawing thousands of names from a large population millions of times is quite computationally intensive, I programmed the Monte Carlo sampling in *C*, using the function *gsl_ran_choose* from the *GNU Scientific Library 1.14 (* www.gnu.org/gsl *)*.

One could have taken a different approach and computed directly the probability of observing any given number of names in a sample of size 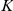. Interestingly, this seemingly simple problem has been solved, but the solution is computationally inapplicable for such a large dataset with current computing power. In fact, the formula [7] involves a sum over all the subsets of size 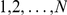 of the 27,220 unique names in the dataset. A multinomial approximation exists [6], but also here the use is questionable, given the large size of some disciplines (*MED* constitutes about 17.5% of the population). One could approximate the probability of the number of non-observed last names using a Poisson distribution [6]. However, even implementing the correct approximation for the 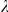 of the Poisson does not give satisfactory results for this particular dataset, probably because of the extreme skewness of the name distribution. This is why I took a simpler, albeit computationally expensive, approach to the problem. The procedure was repeated for micro-sectors (Supplementary Table 1 in Supporting Information S1).

### Logistic Regression Models

One can imagine the space of name-sharing among researchers as a network where two researchers are connected if they share the last name. In each field there are 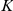 researchers, generating a network containing up to 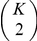 connections. Logistic regression models are one of the standard way to classify and predict the affiliation to two classes. In this case, for each couple of researchers we want to predict if they will share the last name based on different covariates: i) Do the two professors work in the same institution? ii) Do they work in the same micro-sector? iii) At what latitude do they work?

Before we can answer whether any of the covariates does influence the probability of sharing the last name, we need to account for the geographic distribution of the names. If we take two researchers, 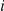 and 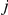, 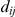 is the distance between their institution based on geographical coordinates computed using the standard ellipsoid model of the Earth. The distance does not therefore account for travel time, geographic barriers and so forth. Two researchers working in the same city have distance zero. The baseline logistic model I used is the following:
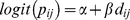
(1)that is to say, there is a baseline probability that 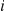 and 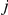 share the last name (controlled by the intercept 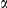), and the probability of connection is enhanced (positive 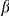) or depressed (negative 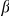) the farther apart the two professors are. In all disciplines but 4 (Table 1 and Supplementary Table 3 in Supporting Information S1), I found significant and negative 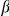
s.

From this baseline “geographic” model, I built three models to account for the different covariates. If 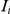 and 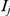 are the institutions 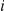 and 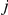 belong to, the second model can be written as:
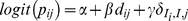
(2)where 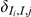 is a Kronecker delta that takes value 1 whenever 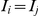 and 0 otherwise. Positive 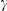 stand for an increase in probability when the two academics are in the same institution. I found statistically significant positive 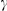
s for most of the disciplines (Table 1 and Supplementary Table 4 in Supporting Information S1).

Similarly, the model accounting for micro-sector can be written as:
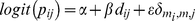
(3)where 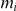 is the micro-sector of researcher 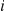. In 10 cases I found significant positive 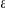
s (Table 1 and Supplementary Table 5 in Supporting Information S1).

Finally, the model:
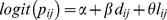
(4)where 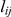 is the average latitude (in degrees) of academics 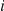 and 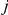, is the statistical model I used to assess the significance of the latitudinal gradient. In 19 cases I found statistically significant negative 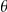
s, standing for an increase in the probability of sharing names when one moves from north to south (Table 1 and Supplementary Table 6 in Supporting Information S1).

The logistic regression analysis was performed in *R* using the standard *glm* function. However, for the three largest disciplinary sectors, the use of *glm* is prohibitive in terms of memory use (analyzing *MED* requires building a matrix with more than 58 millions rows). In these cases, the package *biglm* (*cran.r-project.org/web/packages/biglm*), which provides general linear models for large datasets, was used.

## Supporting Information

Supporting Information S1 (PDF)
