# Supplementary material for: Measuring Nepotism through Shared Last Names: The Case of Italian Academia
Source: PLoS One. 2011 Aug 3;6(8):e21160. doi: 10.1371/journal.pone.0021160 (PMC3149595; doi:10.1371/journal.pone.0021160)
Supplement: Supporting Information S1 — (PDF) [file pone.0021160.s001.pdf]

Measuring Nepotism Through Shared Last Names: the Case of Italian Academia
– Supporting Information –

Stefano Allesina[1,*]

1 Department of Ecology & Evolution, Computation Institute, University of Chicago, Chicago, IL, USA.

∗ **E-mail: Corresponding sallesina@uchicago.edu**

Supplementary Tables

Table S 1. Abbreviations for macro-sectors

Macro-sector	Abbreviation	# micro	# micro $p < 0.05$
Industrial Engineering	ING-IND	35	2
Law	IUS	21	3
Medical sciences	MED	50	15
Geography	M-GGR	2	1
Pedagogy	M-PED	4	3
Agriculture	AGR	20	2
Civil Engineering	ICAR	22	4
Mathematics	MAT	9	2
Chemistry	CHIM	12	2
History	M-STO	9	0
Earth sciences	GEO	12	0
Philosophy	M-FIL	8	1
Statistics	SECS-S	6	0
Political sciences	SPS	14	0
Veterinary	VET	10	2
Life sciences	BIO	19	4
Informatics	INF	1	0
Physics	FIS	8	0
Economics	SECS-P	13	2
Philology	L-FIL-LET	15	2
Physical education	M-EDF	2	0
Electronic Engineering	ING-INF	7	0
Art history	L-ART	8	0
Archeology	L-ANT	10	0
Near eastern studies	L-OR	23	0
Psychology	M-PSI	8	0
Demography & ethnology	M-DEA	1	0
Linguistics	L-LIN	21	0

For each macro-sector, I report the abbreviation used in the following tables as well as the number of micro-sectors and the number of micro-sector yielding a $p < 0.05$ (Table S2).

Table S 2. Results for micro-sectors.

Description	Micro	People	Names	Expected	p-value
Nutrizione e alimentazione animale	AGR18	95	90	94.413	$<10^{-3}$
Misure meccaniche e termiche	ING-IND12	46	43	45.862	$<10^{-3}$
Filosofia e teoria dei linguaggi	M-FIL05	115	109	114.142	$<10^{-3}$
Geografia economico-politica	M-GGR02	141	134	139.718	$<10^{-3}$
Malattie apparato visivo	MED30	266	252	261.564	$<10^{-3}$
Otorinolaringoiatria	MED31	160	147	158.357	$<10^{-3}$
Neuroradiologia	MED37	45	42	44.868	$<10^{-3}$
Farmacologia	BIO14	681	637	654.366	0.001
Strade, ferrovie e aeroporti	ICAR04	100	95	99.350	0.001
Didattica e pedagogia speciale	M-PED03	172	164	170.107	0.001
Chirurgia generale	MED18	1013	933	957.438	0.001
Igiene generale e applicata	MED42	361	342	353.008	0.001
Economia aziendale	SECS-P07	746	694	714.447	0.001
Nefrologia	MED14	115	110	114.142	0.002
Malattie del sangue	MED15	183	175	180.861	0.002
Chimica degli alimenti	CHIM10	66	63	65.716	0.003
Geotecnica	ICAR07	145	139	143.648	0.003
Diritto privato	IUS01	746	697	714.435	0.003
Diritto amministrativo	IUS10	446	423	434.029	0.003
Malattie dell'apparato respiratorio	MED10	129	124	127.925	0.006
Chirurgia toracica	MED21	72	69	71.660	0.006
Economia ed estimo rurale	AGR01	340	325	332.876	0.008
Anatomia degli animali domestici	VET01	106	102	105.269	0.008
Architettura degli interni e allestimento	ICAR16	46	44	45.862	0.009
Neurochirurgia	MED27	137	132	135.789	0.009
Clinica medica veterinaria	VET08	109	105	108.228	0.009
Filologia classica	L-FIL-LET05	81	78	80.570	0.011
Medicina del lavoro	MED44	166	160	164.233	0.011
Fisica matematica	MAT07	332	318	325.195	0.012
Ingegneria sanitaria	ICAR03	92	89	91.447	0.02
Biochimica	BIO10	906	846	860.751	0.021
Diritto privato comparato	IUS02	154	149	152.475	0.021
Medicina fisica e riabilitativa	MED34	59	57	58.772	0.023
Biologia farmaceutica	BIO15	97	94	96.388	0.026
Economia degli intermediari finanziari	SECS-P11	255	246	250.914	0.026
Malattie dell'apparato cardiovascolare	MED11	258	249	253.821	0.03
Ginecologia e ostetricia	MED40	440	421	428.329	0.032
Critica letteraria e letterature comparate	L-FIL-LET14	103	100	102.310	0.035
Pedagogia generale e sociale	M-PED01	326	314	319.433	0.039
Chimica industriale	CHIM04	139	135	137.753	0.04
Storia della pedagogia	M-PED02	106	103	105.269	0.04
Malattie apparato locomotore	MED33	221	214	217.904	0.042
Fisiologia	BIO09	616	585	593.938	0.044
Analisi matematica	MAT05	862	809	820.718	0.046
Chimica industriale e tecnologica	ING-IND27	72	70	71.661	0.047
Anatomia umana	BIO16	352	339	344.386	0.05
Urbanistica	ICAR21	227	220	223.745	0.051
Storia della filosofia	M-FIL06	294	284	288.619	0.051
Anatomia patologica	MED08	315	304	308.851	0.053
Malattie odontostomatologiche	MED28	423	406	412.174	0.053

continued on next page

Description	Micro	People	Names	Expected	p-value
Fisiologia vegetale	BIO04	113	110	112.172	0.054
Campi elettromagnetici	ING-INF02	177	172	174.999	0.055
Pedologia	AGR14	30	29	29.941	0.057
Idraulica	ICAR01	148	144	146.588	0.058
Sistemi elettrici per l'energia	ING-IND33	115	112	114.144	0.058
Scienza delle finanze	SECS-P03	205	199	202.331	0.058
Restauro	ICAR19	149	145	147.570	0.06
Chirurgia vascolare	MED22	116	113	115.128	0.06
Malattie cutanee e veneree	MED35	179	174	176.949	0.06
Istituzioni di diritto pubblico	IUS09	339	327	331.916	0.061
Diritto dell'economia	IUS05	78	76	77.603	0.062
Endocrinologia	MED13	256	248	251.885	0.062
Fisica tecnica industriale	ING-IND10	184	179	181.836	0.072
Storia delle religioni	M-STO06	34	33	33.924	0.073
Psichiatria	MED25	212	206	209.146	0.073
Geometria	MAT03	497	476	482.287	0.075
Economia e gestione delle imprese	SECS-P08	556	531	537.811	0.077
Didattica e storia della fisica	FIS08	36	35	35.915	0.081
Demografia	SECS-S04	85	83	84.530	0.083
Scienza delle costruzioni	ICAR08	351	339	343.427	0.088
Matematiche complementari	MAT04	89	87	88.484	0.097
Zootecnica speciale	AGR19	165	161	163.255	0.103
Chimica farmaceutica	CHIM08	465	447	452.035	0.112
Geografia fisica e geomorfologia	GEO04	134	131	132.841	0.114
Psicologia dello sviluppo e psicologia dell'educazione	M-PSI04	201	196	198.428	0.122
Produzione edilizia	ICAR11	45	44	44.868	0.123
Diritto agrario	IUS03	45	44	44.868	0.123
Medicina legale	MED43	258	251	253.822	0.134
Urologia	MED24	174	170	172.061	0.135
Architettura tecnica	ICAR10	142	139	140.699	0.145
Diritto canonico e diritto ecclesiastico	IUS11	142	139	140.702	0.145
Neurologia	MED26	409	395	398.848	0.151
Zoologia	BIO05	288	280	282.832	0.156
Lingua e traduzione	L-LIN04	145	142	143.647	0.158
Malattie infettive degli animali domestici	VET05	104	102	103.299	0.158
Telecomunicazioni	ING-INF03	334	324	327.117	0.162
Neuropsichiatria infantile	MED39	105	103	104.284	0.162
Diritto commerciale	IUS04	430	415	418.825	0.163
Farmaceutico tecnologico applicativo	CHIM09	212	207	209.148	0.164
Scienze tecniche dietetiche applicate	MED49	53	52	52.818	0.165
Ricerca operativa	MAT09	147	144	145.608	0.167
Fisica sperimentale	FIS01	945	889	896.098	0.173
Fisica tecnica ambientale	ING-IND11	184	180	181.836	0.177
Istologia	BIO17	185	181	182.817	0.18
Informatica	INF01	834	789	795.169	0.182
Impianti chimici	ING-IND25	110	108	109.216	0.187
Bioingegneria elettronica e informatica	ING-INF06	110	108	109.216	0.187
Metodi matematici dell'economia e delle scienze attuariali e finanziarie	SECS-S06	419	405	408.367	0.189
Storia delle dottrine politiche	SPS02	153	150	151.494	0.194
Fisica per il sistema terra e per il mezzo circumterrestre	FIS06	58	57	57.781	0.195
Scienze merceologiche	SECS-P13	112	110	111.186	0.197
Arboricoltura generale e coltivazioni arboree	AGR03	154	151	152.476	0.199
Paleontologia e paleoecologia	GEO01	113	111	112.172	0.202
Scienza e tecnologia dei materiali	ING-IND22	250	244	246.071	0.207

continued on next page

Description	Micro	People	Names	Expected	p-value
Musicologia e storia della musica	L-ART07	115	113	114.142	0.213
Chirurgia pediatrica e infantile	MED20	61	60	60.757	0.214
Storia contemporanea	M-STO04	510	491	494.550	0.217
Ingegneria economico-gestionale	ING-IND35	193	189	190.626	0.219
Metodi e didattiche delle attivita' motorie	M-EDF01	62	61	61.749	0.22
Audiologia	MED32	62	61	61.748	0.221
Ecologia	BIO07	225	220	221.798	0.223
Malattie infettive	MED17	159	156	157.376	0.225
Costruzioni e strutture aerospaziali	ING-IND04	63	62	62.741	0.227
Patologia vegetale	AGR12	160	157	158.357	0.23
Disegno	ICAR17	255	249	250.915	0.231
Diritto costituzionale	IUS08	255	249	250.912	0.232
Elettrotecnica	ING-IND31	198	194	195.501	0.244
Chirurgia cardiaca	MED23	121	119	120.052	0.246
Sociologia giuridica, della devianza e mutamento sociale	SPS12	67	66	66.708	0.252
Clinica ostetrica e ginecologia veterinaria	VET10	67	66	66.707	0.252
Diritto tributario	IUS12	200	196	197.458	0.254
Storia delle istituzioni politiche	SPS03	68	67	67.698	0.259
Gastroenterologia	MED12	166	163	164.236	0.261
Microbiologia e microbiologia clinica	MED07	357	347	349.179	0.264
Antropologia	BIO08	69	68	68.689	0.265
Misure elettriche ed elettroniche	ING-INF07	125	123	123.991	0.267
Economia applicata	SECS-P06	167	164	165.213	0.267
Archeologia classica	L-ANT07	167	164	165.211	0.268
Storia della scienza e delle tecniche	M-STO05	70	69	69.680	0.272
Estimo	ICAR22	71	70	70.671	0.278
Pedagogia sperimentale	M-PED04	71	70	70.672	0.278
Geografia	M-GGR01	236	231	232.485	0.279
Elettronica	ING-INF01	360	350	352.047	0.28
Diritto del lavoro	IUS07	338	329	330.957	0.28
Economia politica	SECS-P01	847	803	807.042	0.283
Tecnologie e sistemi di lavorazione	ING-IND16	170	167	168.148	0.284
Lingua e letterature anglo-americane	L-LIN11	73	72	72.652	0.291
Paleografia	M-STO09	73	72	72.653	0.291
Sistemi di elaborazione delle informazioni	ING-INF05	696	665	668.260	0.294
Macchine a fluido	ING-IND08	172	169	170.107	0.295
Idraulica agraria e sistemazioni idraulico-forestali	AGR08	74	73	73.643	0.298
Geologia stratigrafica e sedimentologica	GEO02	173	170	171.085	0.301
Patologia generale	MED04	560	539	541.555	0.308
Probabilita' e statistica matematica	MAT06	132	130	130.874	0.31
Filologia della letteratura italiana	L-FIL-LET13	76	75	75.623	0.312
Linguistica italiana	L-FIL-LET12	177	174	174.998	0.324
Sociologia dei processi culturali e comunicativi	SPS08	301	294	295.368	0.336
Fisiologia veterinaria	VET02	80	79	79.584	0.338
Finanza aziendale	SECS-P09	81	80	80.573	0.345
Museologia e critica artistica e del restauro	L-ART04	81	80	80.572	0.346
Sociologia generale	SPS07	435	422	423.583	0.36
Diritto penale	IUS17	306	299	300.188	0.365
Chimica analitica	CHIM01	280	274	275.106	0.366
Genetica medica	MED03	142	140	140.700	0.372
Chirurgia plastica	MED19	85	84	84.529	0.373
Microbiologia agraria	AGR16	143	141	141.681	0.378
Storia romana	L-ANT03	145	143	143.647	0.39
Chimica generale e inorganica	CHIM03	576	555	556.558	0.393

continued on next page

Description	Micro	People	Names	Expected	p-value
Sistemi per l'energia e l'ambiente	ING-IND09	89	88	88.485	0.399
Lingua e traduzione	L-LIN12	337	329	329.997	0.402
Disegno e metodi dell'ingegneria industriale	ING-IND15	90	89	89.472	0.407
Automatica	ING-INF04	287	281	281.864	0.408
Impianti industriali meccanici	ING-IND17	148	146	146.589	0.41
Clinica chirurgica veterinaria	VET09	93	92	92.437	0.426
Geologia applicata	GEO05	152	150	150.514	0.435
Diagnostica per immagini e radioterapia	MED36	366	357	357.789	0.437
Meccanica applicata alle macchine	ING-IND13	197	194	194.530	0.446
Genetica agraria	AGR07	96	95	95.400	0.447
Lingua e letteratura latina	L-FIL-LET04	265	260	260.596	0.448
Pediatria generale e specialistica	MED38	470	456	456.768	0.453
Letteratura italiana	L-FIL-LET10	433	421	421.681	0.458
Trasporti	ICAR05	99	98	98.363	0.467
Petrologia e petrografia	GEO07	101	100	100.337	0.481
Anatomia comparata e citologia	BIO06	239	235	235.397	0.482
Statistica economica	SECS-S03	160	158	158.356	0.486
Diritto processuale civile	IUS15	205	202	202.327	0.497
Lingua e letteratura greca	L-FIL-LET02	164	162	162.274	0.511
Mineralogia	GEO06	106	105	105.271	0.513
Letteratura spagnola	L-LIN05	106	105	105.271	0.513
Tecnica delle costruzioni	ICAR09	333	326	326.164	0.523
Storia dell'arte moderna	L-ART02	166	164	164.234	0.523
Storia moderna	M-STO02	333	326	326.156	0.524
Psicologia sociale	M-PSI05	167	165	165.215	0.529
Anestesiologia	MED41	280	275	275.108	0.537
Diritto processuale penale	IUS16	212	209	209.151	0.539
Biochimica clinica e biologia molecolare clinica	BIO12	169	167	167.172	0.542
Microbiologia generale	BIO19	111	110	110.200	0.546
Convertitori, macchine e azionamenti elettrici	ING-IND32	114	113	113.157	0.565
Lingua e traduzione	L-LIN14	114	113	113.158	0.565
Politica economica	SECS-P02	365	357	356.841	0.566
Storia del diritto medievale e moderno	IUS19	175	173	173.042	0.578
Patologia clinica	MED05	117	116	116.114	0.583
Analisi numerica	MAT08	257	253	252.853	0.59
Storia medievale	M-STO01	221	218	217.907	0.593
Tecnica e pianificazione urbanistica	ICAR20	178	176	175.973	0.597
Biologia molecolare	BIO11	222	219	218.878	0.599
Tecnologia dell'architettura	ICAR12	222	219	218.880	0.599
Composizione architettonica e urbana	ICAR14	478	465	464.336	0.605
Astronomia e astrofisica	FIS05	182	180	179.882	0.62
Entomologia generale e applicata	AGR11	124	123	123.006	0.625
Diritto pubblico comparato	IUS21	125	124	123.989	0.631
Chimica fisica	CHIM02	444	433	432.124	0.636
Botanica generale	BIO01	126	125	124.973	0.637
Lingua e traduzione	L-LIN07	127	126	125.956	0.643
Psicologia clinica	M-PSI08	187	185	184.770	0.648
Agronomia e coltivazioni erbacee	AGR02	188	186	185.746	0.653
Oncologia medica	MED06	129	128	127.925	0.653
Fisica nucleare e subnucleare	FIS04	136	135	134.806	0.692
Scienze e tecnologie alimentari	AGR15	198	196	195.503	0.706
Filosofia del diritto	IUS20	242	239	238.312	0.707
Botanica ambientale e applicata	BIO03	142	141	140.702	0.721
Fisica applicata (a beni culturali, ambientali, biologia e medicina)	FIS07	314	309	307.886	0.725

continued on next page

Description	Micro	People	Names	Expected	p-value
Statistica	SECS-S01	443	433	431.180	0.737
Costruzioni idrauliche e marittime e idrologia	ICAR02	205	203	202.331	0.74
Scienza politica	SPS04	205	203	202.329	0.74
Storia economica	SECS-P12	208	206	205.251	0.755
Genetica	BIO18	209	207	206.227	0.758
Storia dell'architettura	ICAR18	253	250	248.974	0.76
Letteratura inglese	L-LIN10	293	289	287.654	0.775
Chimica organica	CHIM06	652	631	627.450	0.787
Fisica della materia	FIS03	457	447	444.454	0.797
Slavistica	L-LIN21	162	161	160.316	0.809
Filosofia morale	M-FIL03	225	223	221.798	0.823
Biologia applicata	BIO13	271	268	266.403	0.832
Diritto romano e diritti dell'antichita'	IUS18	273	270	268.338	0.839
Medicina interna	MED09	1020	971	963.728	0.85
Progettazione meccanica e costruzione di macchine	ING-IND14	176	175	174.017	0.858
Algebra	MAT02	178	177	175.976	0.863
Glottologia e linguistica	L-LIN01	288	285	282.831	0.884
Filosofia teoretica	M-FIL01	187	186	184.768	0.889
Fondamenti chimici delle tecnologie	CHIM07	193	192	190.628	0.903
Psicologia generale	M-PSI01	300	297	294.405	0.913
Letteratura francese	L-LIN03	198	197	195.504	0.914
Fisica teorica, modelli e metodi matematici	FIS02	344	340	336.717	0.927
Orticoltura e floricoltura	AGR04	56	56	55.795	1
Assestamento forestale e selvicoltura	AGR05	71	71	70.672	1
Tecnologia del legno e utilizzazioni forestali	AGR06	17	17	16.982	1
Meccanica agraria	AGR09	93	93	92.438	1
Costruzioni rurali e territorio agroforestale	AGR10	73	73	72.652	1
Chimica agraria	AGR13	136	136	134.807	1
Zootecnica generale e miglioramento genetico	AGR17	81	81	80.572	1
Zoocolture	AGR20	51	51	50.830	1
Botanica sistematica	BIO02	119	119	118.085	1
Scienza e tecnologia dei materiali polimerici	CHIM05	2	2	2.000	1
Chimica e biotecnologia delle fermentazioni	CHIM11	38	38	37.906	1
Chimica dell'ambiente e dei beni culturali	CHIM12	62	62	61.750	1
Geologia strutturale	GEO03	101	101	100.337	1
Geochimica e vulcanologia	GEO08	89	89	88.483	1
Georisorse minerarie e app. mineralogico-petrografiche	GEO09	74	74	73.642	1
Geofisica della terra solida	GEO10	80	80	79.583	1
Geofisica applicata	GEO11	50	50	49.837	1
Oceanografia e fisica dell'atmosfera	GEO12	23	23	22.966	1
Topografia e cartografia	ICAR06	115	115	114.143	1
Disegno industriale	ICAR13	147	147	145.606	1
Architettura del paesaggio	ICAR15	35	35	34.920	1
Architettura navale	ING-IND01	22	22	21.969	1
Costruzioni e impianti navali e marini	ING-IND02	18	18	17.980	1
Meccanica del volo	ING-IND03	28	28	27.949	1
Impianti e sistemi aerospaziali	ING-IND05	33	33	32.929	1
Fluidodinamica	ING-IND06	67	67	66.707	1
Propulsione aerospaziale	ING-IND07	30	30	29.942	1
Fisica dei reattori nucleari	ING-IND18	12	12	11.991	1
Impianti nucleari	ING-IND19	44	44	43.874	1
Misure e strumentazione nucleari	ING-IND20	13	13	12.990	1
Metallurgia	ING-IND21	91	91	90.461	1
Chimica fisica applicata	ING-IND23	20	20	19.974	1

continued on next page

Description	Micro	People	Names	Expected	p-value
Principi di ingegneria chimica	ING-IND24	85	85	84.528	1
Teoria dello sviluppo dei processi chimici	ING-IND26	35	35	34.920	1
Ingegneria e sicurezza degli scavi	ING-IND28	27	27	26.953	1
Ingegneria delle materie prime	ING-IND29	15	15	14.986	1
Idrocarburi e fluidi del sottosuolo	ING-IND30	9	9	8.995	1
Bioingegneria industriale	ING-IND34	60	60	59.766	1
Diritto della navigazione	IUS06	54	54	53.810	1
Diritto internazionale	IUS13	302	302	296.338	1
Diritto dell'unione europea	IUS14	77	77	76.612	1
Preistoria e protostoria	L-ANT01	60	60	59.765	1
Storia greca	L-ANT02	103	103	102.312	1
Numismatica	L-ANT04	23	23	22.966	1
Papirologia	L-ANT05	20	20	19.975	1
Etruscologia e antichita' italiche	L-ANT06	33	33	32.930	1
Archeologia cristiana e medievale	L-ANT08	78	78	77.603	1
Topografia antica	L-ANT09	45	45	44.867	1
Metodologie della ricerca archeologica	L-ANT10	30	30	29.942	1
Storia dell'arte medievale	L-ART01	87	87	86.507	1
Storia dell'arte contemporanea	L-ART03	96	96	95.400	1
Discipline dello spettacolo	L-ART05	119	119	118.083	1
Cinema, fotografia e televisione	L-ART06	135	135	133.825	1
Etnomusicologia	L-ART08	16	16	15.984	1
Civilta' egee	L-FIL-LET01	11	11	10.992	1
Filologia italica, illirica, celtica	L-FIL-LET03	1	1	1.000	1
Letteratura cristiana antica	L-FIL-LET06	49	49	48.843	1
Civilta' bizantina	L-FIL-LET07	37	37	36.911	1
Letteratura latina medievale e umanistica	L-FIL-LET08	57	57	56.788	1
Filologia e linguistica romanza	L-FIL-LET09	112	112	111.186	1
Letteratura italiana contemporanea	L-FIL-LET11	146	146	144.626	1
Filologia germanica	L-FIL-LET15	68	68	67.698	1
Didattica delle lingue moderne	L-LIN02	67	67	66.706	1
Lingua e letterature ispano-americane	L-LIN06	35	35	34.920	1
Letterature portoghese e brasiliana	L-LIN08	24	24	23.963	1
Lingua e traduzione	L-LIN09	10	10	9.994	1
Letteratura tedesca	L-LIN13	140	140	138.736	1
Lingue e letterature nordiche	L-LIN15	7	7	6.997	1
Lingua e letteratura nederlandese	L-LIN16	4	4	3.999	1
Lingua e letteratura romena	L-LIN17	11	11	10.993	1
Lingua e letteratura albanese	L-LIN18	11	11	10.993	1
Filologia ugro-finnica	L-LIN19	10	10	9.994	1
Lingua e letteratura neogreca	L-LIN20	11	11	10.992	1
Storia del vicino oriente antico	L-OR01	11	11	10.993	1
Egittologia e civilta' copta	L-OR02	15	15	14.986	1
Assiriologia	L-OR03	8	8	7.996	1
Anatolistica	L-OR04	8	8	7.996	1
Archeologia e storia dell'arte del vicino oriente antico	L-OR05	13	13	12.989	1
Archeologia fenicio-punica	L-OR06	9	9	8.995	1
Semitistica-lingue e letterature dell'Etiopia	L-OR07	8	8	7.996	1
Ebraico	L-OR08	14	14	13.988	1
Lingue e letterature dell'Africa	L-OR09	8	8	7.996	1
Storia dei paesi islamici	L-OR10	23	23	22.966	1
Archeologia e storia dell'arte musulmana	L-OR11	5	5	4.999	1
Lingua e letteratura araba	L-OR12	40	40	39.896	1
Armenistica, caucasologia, mongolistica e turcologia	L-OR13	8	8	7.996	1

continued on next page

Description	Micro	People	Names	Expected	p-value
Filologia, religioni e storia dell'Iran	L-OR14	7	7	6.997	1
Lingua e letteratura persiana	L-OR15	9	9	8.995	1
Archeologia e storia dell'arte dell'India e dell'Asia centrale	L-OR16	7	7	6.997	1
Filosofie, religioni e storia dell'India e dell'Asia centrale	L-OR17	7	7	6.997	1
Indologia e tibetologia	L-OR18	15	15	14.986	1
Lingue e Letterature moderne del subcontinente indiano	L-OR19	7	7	6.997	1
Archeologia, storia dell'arte e filosofie dell'Asia orientale	L-OR20	8	8	7.996	1
Lingue e Letterature della Cina e dell'Asia sud-orientale	L-OR21	49	49	48.843	1
Lingue e letterature del Giappone e della Corea	L-OR22	27	27	26.953	1
Storia dell'Asia orientale e sud-orientale	L-OR23	11	11	10.992	1
Discipline demoetnoantropologiche	M-DEA01	195	195	192.577	1
Metodi e didattiche delle attivita' sportive	M-EDF02	76	76	75.622	1
Logica e filosofia della scienza	M-FIL02	101	101	100.338	1
Estetica	M-FIL04	102	102	101.323	1
Storia della filosofia antica	M-FIL07	51	51	50.830	1
Storia della filosofia medievale	M-FIL08	50	50	49.837	1
Psicobiologia e psicologia fisiologica	M-PSI02	112	112	111.187	1
Psicometria	M-PSI03	74	74	73.642	1
Psicologia del lavoro e delle organizzazioni	M-PSI06	86	86	85.518	1
Psicologia dinamica	M-PSI07	125	125	123.988	1
Storia dell'Europa orientale	M-STO03	41	41	40.891	1
Storia del cristianesimo e delle chiese	M-STO07	82	82	81.563	1
Archivistica, bibliografia e biblioteconomia	M-STO08	89	89	88.485	1
Logica matematica	MAT01	37	37	36.911	1
Statistica medica	MED01	100	100	99.349	1
Storia della medicina	MED02	35	35	34.921	1
Reumatologia	MED16	110	110	109.214	1
Chirurgia maxillofacciale	MED29	81	81	80.573	1
Scienze infermieristiche generali, cliniche e pediatriche	MED45	34	34	33.925	1
Scienze tecniche di medicina e di laboratorio	MED46	89	89	88.485	1
Scienze infermieristiche ostetrico-ginecologiche	MED47	12	12	11.991	1
Scienze infermieristiche e tecniche neuro-psichiatriche e riabilitative	MED48	16	16	15.984	1
Scienze tecniche mediche applicate	MED50	86	86	85.519	1
Storia del pensiero economico	SECS-P04	45	45	44.868	1
Econometria	SECS-P05	71	71	70.671	1
Organizzazione aziendale	SECS-P10	148	148	146.588	1
Statistica per la ricerca sperimentale e tecnologica	SECS-S02	30	30	29.942	1
Statistica sociale	SECS-S05	75	75	74.634	1
Filosofia politica	SPS01	118	118	117.096	1
Storia e istituzioni delle Americhe	SPS05	29	29	28.946	1
Storia delle relazioni internazionali	SPS06	68	68	67.698	1
Sociologia dei processi economici e del lavoro	SPS09	164	164	162.277	1
Sociologia dell'ambiente e del territorio	SPS10	77	77	76.613	1
Sociologia dei fenomeni politici	SPS11	52	52	51.824	1
Storia e istituzioni dell'Africa	SPS13	36	36	35.916	1
Storia e istituzioni dell'Asia	SPS14	19	19	18.977	1
Patologia generale e anatomia patologica veterinaria	VET03	91	91	90.461	1
Ispezione degli alimenti di origine animale	VET04	80	80	79.583	1
Parassitologia e malattie parassitarie degli animali	VET06	71	71	70.671	1
Farmacologia e tossicologia veterinaria	VET07	46	46	45.862	1

Table S 3. Results of Logistic Regression: Eq. 1

Discipline	α	p_α	β	p_β
AGR	-7.91	0	-0.0029	$2.82 \cdot 10^{-32}$
BIO	-8.27	0	-0.0015	$7.16 \cdot 10^{-52}$
CHIM	-8.16	0	-0.0021	$1.41 \cdot 10^{-32}$
FIS	-8.56	0	-0.0008	$2.13 \cdot 10^{-5}$
GEO	-8.16	0	-0.0020	$1.27 \cdot 10^{-5}$
ICAR	-8.33	0	-0.0011	$5.4 \cdot 10^{-21}$
INF	-7.73	0	-0.0020	$5.07 \cdot 10^{-5}$
ING-IND	-8.17	0	-0.0013	$1.32 \cdot 10^{-21}$
ING-INF	-8.38	0	-0.0017	$5.57 \cdot 10^{-10}$
IUS	-8.28	0	-0.0014	$8.62 \cdot 10^{-44}$
L-ANT	-7.93	0	-0.0034	0.0003
L-ART	-8.84	0	0.0000	0.99
L-FIL-LET	-8.39	0	-0.0014	$2.49 \cdot 10^{-6}$
L-LIN	-8.97	0	-0.0009	0.0021
L-OR	-8.79	0	-0.0013	0.5356
M-DEA	-23.57	0.98	0	1
M-EDF	-7.73	0	-0.0023	0.5173
M-FIL	-8.21	0	-0.0013	0.0018
M-GGR	-7.02	0	-0.0035	0.0019
M-PED	-7.47	0	-0.0031	$2.33 \cdot 10^{-5}$
M-PSI	-8.41	0	-0.0016	0.0003
M-STO	-8.43	0	-0.0010	0.0032
MAT	-8.25	0	-0.0017	$1.53 \cdot 10^{-16}$
MED	-8.43	0	-0.0011	$2.89 \cdot 10^{-125}$
SECS-P	-8.51	0	-0.0010	$2.03 \cdot 10^{-14}$
SECS-S	-7.99	0	-0.0026	$7.21 \cdot 10^{-8}$
SPS	-8.17	0	-0.0018	$2.11 \cdot 10^{-10}$
VET	-7.88	0	-0.0030	$1.28 \cdot 10^{-5}$

Results for the logistic regression introduced in Equation 1. For each coefficient, I report the fitted values and the associated probabilities. α is the intercept, β is the coefficient accounting for geographic effects.

Table S 4. Results of Logistic Regression: Eq. 2

Discipline	α	p_α	β	p_β	γ	p_γ
AGR	-8.206	0	-0.0022	0	0.761	$1.36 \cdot 10^{-6}$
BIO	-8.417	0	-0.0012	0	0.711	0
CHIM	-8.419	0	-0.0015	0	0.980	0
FIS	-8.724	0	-0.0005	0.011	0.868	$4.85 \cdot 10^{-6}$
GEO	-8.132	0	-0.0020	$4.38 \cdot 10^{-5}$	-0.150	0.739
ICAR	-8.537	0	-0.0007	$1.67 \cdot 10^{-8}$	0.684	0
INF	-7.787	0	-0.0019	0.0004	0.236	0.566
ING-IND	-8.488	0	-0.0007	$1.87 \cdot 10^{-6}$	0.976	0
ING-INF	-8.534	0	-0.0013	$1.47 \cdot 10^{-6}$	0.667	0.002
IUS	-8.482	0	-0.0010	0	1.013	0
L-ANT	-8.212	0	-0.0027	0.008	0.873	0.130
L-ART	-8.968	0	0.0002	0.710	0.845	0.191
L-FIL-LET	-8.554	0	-0.0011	0.001	0.821	0.002
L-LIN	-9.067	0	-0.0007	0.019	0.606	0.049
L-OR	-8.166	0	-0.0027	0.285	-6.400	0.619
M-DEA	-23.566	0.984	0.0000	1	0.000	1
M-EDF	-7.434	$1.28 \cdot 10^{-9}$	-0.0031	0.448	-6.132	0.815
M-FIL	-8.390	0	-0.0009	0.032	0.970	0.008
M-GGR	-7.278	0	-0.0029	0.017	0.856	0.243
M-PED	-8.149	0	-0.0016	0.041	1.810	$2.05 \cdot 10^{-5}$
M-PSI	-8.495	0	-0.0015	0.004	0.329	0.357
M-STO	-8.529	0	-0.0008	0.024	0.556	0.102
MAT	-8.400	0	-0.0014	$1.25 \cdot 10^{-10}$	0.712	$4.51 \cdot 10^{-5}$
MED	-8.621	0	-0.0007	0	0.773	0
SECS-P	-8.710	0	-0.0006	$6.06 \cdot 10^{-6}$	1.184	0
SECS-S	-8.272	0	-0.0019	0.0001	1.019	0.001
SPS	-8.294	0	-0.0015	$2.34 \cdot 10^{-7}$	0.583	0.018
VET	-8.967	0	-0.0006	0.421	1.824	$7.3 \cdot 10^{-6}$

Results for the logistic regression introduced in Equation 2. For each coefficient, I report the fitted value and the associated probability. α is the intercept, β the coefficient accounting for geographic distance and γ the coefficient accounting for the effect of belonging to the same institution.

Table S 5. Results of Logistic Regression: Eq. 3

Discipline	α	p_α	β	p_β	ϵ	p_ϵ
AGR	-7.945	0	-0.0029	$3.25 \cdot 10^{-32}$	0.445	0.008
BIO	-8.304	0	-0.0015	$9.64 \cdot 10^{-52}$	0.301	$2.23 \cdot 10^{-5}$
CHIM	-8.161	0	-0.0021	$1.43 \cdot 10^{-32}$	0.031	0.779
FIS	-8.550	0	-0.0008	$2.15 \cdot 10^{-5}$	-0.038	0.743
GEO	-8.183	0	-0.0019	$1.31 \cdot 10^{-5}$	0.204	0.504
ICAR	-8.352	0	-0.0011	$6.79 \cdot 10^{-21}$	0.249	0.022
INF	-7.730	0	-0.0020	$5.07 \cdot 10^{-5}$	NA	NS
ING-IND	-8.190	0	-0.0013	$1.46 \cdot 10^{-21}$	0.332	0.019
ING-INF	-8.435	0	-0.0017	$5.33 \cdot 10^{-10}$	0.259	0.063
IUS	-8.303	0	-0.0014	$8.53 \cdot 10^{-44}$	0.250	0.0021
L-ANT	-7.956	$1.18 \cdot 10^{-147}$	-0.0034	0.0004	0.134	0.784
L-ART	-8.795	$7.85 \cdot 10^{-228}$	$-9.41 \cdot 10^{-6}$	0.9875	-0.348	0.453
L-FIL-LET	-8.408	0	0.1401	0.4791	0.140	0.479
L-LIN	-8.992	0	-0.0009	0.002	0.174	0.430
L-OR	-8.721	$1.63 \cdot 10^{-30}$	-0.0013	0.5350	-5.417	0.720
M-DEA	-23.566	0.983	0.0000	1	NA	NS
M-EDF	-7.724	$6.5 \cdot 10^{-9}$	-0.0023	0.5173	-0.005	0.997
M-FIL	-8.243	0	-0.0013	0.0018	0.202	0.404
M-GGR	-7.243	$9.61 \cdot 10^{-51}$	-0.0034	0.0021	0.367	0.437
M-PED	-7.749	$6.96 \cdot 10^{-171}$	-0.0031	$2.16 \cdot 10^{-5}$	0.683	0.019
M-PSI	-8.363	0	-0.0016	0.0003	-0.354	0.267
M-STO	-8.430	0	-0.0010	0.0032	-0.005	0.979
MAT	-8.333	0	-0.0017	$1.36 \cdot 10^{-16}$	0.354	0.0008
MED	-8.447	0	-0.0011	$2.73 \cdot 10^{-125}$	0.298	$1.39 \cdot 10^{-9}$
SECS-P	-8.565	0	-0.0010	$2.2 \cdot 10^{-14}$	0.347	$2.52 \cdot 10^{-5}$
SECS-S	-7.988	0	-0.0026	$7.21 \cdot 10^{-8}$	-0.010	0.964
SPS	-8.167	0	-0.0018	$2.07 \cdot 10^{-10}$	-0.055	0.778
VET	-8.067	$3.73 \cdot 10^{-275}$	-0.0030	$1.27 \cdot 10^{-5}$	1.105	0.0004

Results for the logistic regression introduced in Equation 3. For each coefficient, I report the fitted value and the associated probability. α is the intercept, β the coefficient accounting for geographic distance and ϵ the coefficient accounting for the effect of belonging to the same micro-sector.

Table S 6. Results of Logistic Regression: Eq. 4

Discipline	α	p_α	β	p_β	θ	p_θ
AGR	0.714	0.456	-0.0032	$3.24 \cdot 10^{-39}$	-0.201	$6.54 \cdot 10^{-19}$
BIO	-5.044	$1.64 \cdot 10^{-19}$	-0.0017	$2.06 \cdot 10^{-59}$	-0.074	$7.65 \cdot 10^{-9}$
CHIM	-1.429	0.085	-0.0025	$2.5 \cdot 10^{-43}$	-0.154	$7.32 \cdot 10^{-16}$
FIS	-2.147	0.054	-0.0011	$1.75 \cdot 10^{-8}$	-0.147	$1.1 \cdot 10^{-8}$
GEO	-1.996	0.383	-0.0022	$1.44 \cdot 10^{-6}$	-0.142	0.007
ICAR	-0.540	0.381	-0.0013	$2.32 \cdot 10^{-29}$	-0.181	$4.23 \cdot 10^{-36}$
INF	-14.536	0.0002	-0.0016	0.0063	0.152	0.079
ING-IND	-2.891	0.0003	-0.0016	$8.9 \cdot 10^{-29}$	-0.120	$5.15 \cdot 10^{-11}$
ING-INF	-4.525	0.003	-0.0019	$1.94 \cdot 10^{-11}$	-0.087	0.012
IUS	-1.175	0.023	-0.0015	$2.18 \cdot 10^{-55}$	-0.166	$2.0 \cdot 10^{-42}$
L-ANT	-6.821	0.121	-0.0034	0.0003	-0.026	0.800
L-ART	-7.760	0.072	-0.0001	0.9154	-0.024	0.803
L-FIL-LET	-2.117	0.188	-0.0016	$5.51 \cdot 10^{-8}$	-0.145	0.0001
L-LIN	-2.223	0.184	-0.0012	$4.59 \cdot 10^{-5}$	-0.154	$5.84 \cdot 10^{-5}$
L-OR	4.653	0.675	-0.0015	0.4481	-0.313	0.231
M-DEA	-23.566	0.999	0.0000	1	$1.52 \cdot 10^{-18}$	1
M-EDF	-1.631	0.925	-0.0026	0.4757	-0.141	0.727
M-FIL	0.622	0.772	-0.0016	$7.01 \cdot 10^{-5}$	-0.204	$4.66 \cdot 10^{-5}$
M-GGR	8.488	0.062	-0.0035	0.0012	-0.369	0.0009
M-PED	5.474	0.062	-0.0036	$6.92 \cdot 10^{-7}$	-0.299	$1.37 \cdot 10^{-5}$
M-PSI	-11.485	0.0002	-0.0014	0.0046	0.069	0.312
M-STO	-4.430	0.031	-0.0012	0.0008	-0.092	0.052
MAT	0.617	0.548	-0.0021	$8.24 \cdot 10^{-24}$	-0.204	$1.04 \cdot 10^{-17}$
MED	-3.801	$8.86 \cdot 10^{-51}$	-0.0013	$8.67 \cdot 10^{-168}$	-0.107	$4.46 \cdot 10^{-74}$
SECS-P	-1.006	0.205	-0.0014	$5.32 \cdot 10^{-24}$	-0.171	$5.3 \cdot 10^{-21}$
SECS-S	1.507	0.469	-0.0029	$9.75 \cdot 10^{-10}$	-0.220	$6.26 \cdot 10^{-6}$
SPS	-7.102	$1.6 \cdot 10^{-5}$	-0.0019	$4.18 \cdot 10^{-10}$	-0.024	0.515
VET	9.096	0.001	-0.0034	$1.13 \cdot 10^{-7}$	-0.394	$2.19 \cdot 10^{-9}$

Results for the logistic regression introduced in Equation 4. For each coefficient, I report the fitted value and the associated probability. α is the intercept, β the coefficient accounting for geographic distance and θ the coefficient expressing the effect of the average latitude of the professors on their connection probability.

Table S 7. Frequency of Last Name-Sharing

Institution	N. Professors	N. Pairs	N. Same Name	Freq. (x1000)	City	Region
LUM Jean Monnet	35	595	1	1.681	Casamassima	Puglia
Sassari	694	240471	314	1.306	Sassari	Sardegna
Cagliari	1111	616605	764	1.239	Cagliari	Sardegna
Suor Orsola Benincasa	86	3655	4	1.094	Napoli	Campania
Catania	1594	1269621	1333	1.05	Catania	Sicilia
UKE - Enna	93	4278	3	0.701	Enna	Sicilia
della Calabria	835	348195	242	0.695	Arcavacata	Calabria
Messina	1313	861328	598	0.694	Messina	Sicilia
Mediterranea R. Calabria	298	44253	30	0.678	Reggio Calabria	Calabria
Rome Foro Italico	55	1485	1	0.673	Roma	Lazio
Salento	719	258121	155	0.6	Lecce	Puglia
Seconda Univ. Napoli	1044	544446	317	0.582	Napoli	Campania
Cassino	332	54946	30	0.546	Cassino	Lazio
Camerino	314	49141	26	0.529	Camerino	Marche
Modena e Reggio Emilia	856	365940	182	0.497	Modena	Emilia-Romagna
Palermo	1946	1892485	940	0.497	Palermo	Sicilia
Politecnico di Bari	345	59340	29	0.489	Bari	Puglia
LUISS Guido Carli - Roma	91	4095	2	0.488	Roma	Lazio
Urbino Carlo Bo	431	92665	43	0.464	Urbino	Marche
Salerno	998	497503	223	0.448	Salerno	Campania
Sannio di Benevento	191	18145	8	0.441	Benevento	Campania
Napoli Federico II	2831	4005865	1764	0.44	Napoli	Campania
Parma	1017	516636	222	0.43	Parma	Emilia-Romagna
Parthenope di Napoli	338	56953	24	0.421	Napoli	Campania
S. Raffaele Milano	99	4851	2	0.412	Milano	Lombardia
L'Aquila	628	196878	69	0.35	Aquila	Abruzzo
Foggia	371	68635	24	0.35	Foggia	Puglia
Bari	1848	1706628	589	0.345	Bari	Puglia
Genova	1501	1125750	384	0.341	Genova	Liguria
Insubria	388	75078	25	0.333	Varese	Lombardia
Ferrara	651	211575	69	0.326	Ferrara	Emilia-Romagna
Pavia	1044	544446	177	0.325	Pavia	Lombardia
Bergamo	335	55945	18	0.322	Bergamo	Lombardia
Basilicata	323	52003	16	0.308	Potenza	Basilicata
Politecnica delle Marche	541	146070	42	0.288	Ancona	Marche
Pisa	1711	1462905	403	0.275	Pisa	Toscana
Siena	991	490545	134	0.273	Siena	Toscana
Perugia	1233	759528	207	0.273	Perugia	Umbria
Firenze	2188	2392578	627	0.262	Firenze	Toscana
Chieti-Pescara	744	276396	71	0.257	Chieti	Abruzzo
Bologna	3088	4766328	1193	0.25	Bologna	Emilia-Romagna
Brescia	563	158203	39	0.247	Brescia	Lombardia
Catanzaro	224	24976	6	0.24	Catanzaro	Calabria
Lib. Univ. di Bolzano	131	8515	2	0.235	Bolzano	Trentino-Alto Adige
Milano Bicocca	2315	2678455	622	0.232	Milano	Lombardia
Cattolica Milano	1478	1091503	243	0.223	Milano	Lombardia
Politecnico di Torino	868	376278	81	0.215	Torino	Piemonte
Roma Tor Vergata	1566	1225395	255	0.208	Roma	Lazio
Macerata	326	52975	11	0.208	Macerata	Marche
Torino	2110	2224995	455	0.204	Torino	Piemonte

continued on next page

Institution	N. Professors	N. Pairs	N. Same Name	Freq. (x1000)	City	Region
Verona	774	299151	60	0.201	Verona	Veneto
Padova	2199	2416701	482	0.199	Padova	Veneto
Molise	302	45451	9	0.198	Campobasso	Molise
IUAV di Venezia	175	15225	3	0.197	Venezia	Veneto
Piemonte Orientale	394	77421	15	0.194	Vercelli	Piemonte
Campus Bio-Medico Roma	102	5151	1	0.194	Roma	Lazio
Milano Bicocca	924	426426	82	0.192	Milano	Lombardia
Politecnico di Milano	1424	1013176	189	0.187	Milano	Lombardia
Roma La Sapienza	4462	9952491	1745	0.175	Roma	Lazio
S. Anna di Pisa	108	5778	1	0.173	Pisa	Toscana
L'Orientale di Napoli	243	29403	5	0.17	Napoli	Campania
Udine	729	265356	42	0.158	Udine	Friuli-Venezia-Giulia
Roma Tre	939	440391	62	0.141	Roma	Lazio
Trento	581	168490	22	0.131	Trento	Trentino-Alto Adige
Trieste	824	339076	41	0.121	Trieste	Friuli-Venezia-Giulia
Teramo	259	33411	4	0.12	Teramo	Abruzzo
Ca' Foscari Venezia	517	133386	15	0.112	Venezia	Veneto
Tuscia	303	45753	5	0.109	Viterbo	Lazio
Bocconi Milano	285	40470	4	0.099	Milano	Lombardia
Valle d'Aosta	57	1596	0	0	Aosta	Val d'Aosta
LIUC - Castellanza	48	1128	0	0	Castellanza	Lombardia
I.U.S.S. - Pavia	6	15	0	0	Pavia	Lombardia
IULM - Milano	94	4371	0	0	Milano	Lombardia
Scienze Gastronomiche	12	66	0	0	Colorno	Emilia-Romagna
Scuola Normale Pisa	112	6216	0	0	Pisa	Toscana
Scuola IMT - Lucca	12	66	0	0	Lucca	Toscana
Stranieri di Siena	41	820	0	0	Siena	Toscana
SUM - Firenze	9	36	0	0	Firenze	Toscana
Stranieri di Perugia	60	1770	0	0	Perugia	Umbria
Europea di Roma	44	946	0	0	Roma	Lazio
LUMSA	84	3486	0	0	Roma	Lazio
LUSPIO	34	561	0	0	Roma	Lazio
SISSA - Trieste	65	2080	0	0	Trieste	Friuli-Venezia-Giulia
Stranieri Reggio Calabria	2	1	0	0	Reggio Calabria	Calabria

Data used to draw Figure 1. For each institution, I report the number of professors (N), the number of possible pairs of professors ($N(N-1)/2$) and the number of pairs having the same last name. From these, I obtained the frequency of same-name pairs drawn in Figure 1 and reported in the table. Institutions are ordered according to the frequency of share-name pairs.
